# Delayed Hemothorax Following Trauma in a COVID-19 Positive Patient Requiring Video-Assisted Thoracoscopic Surgery: A Case Report

**DOI:** 10.7759/cureus.91791

**Published:** 2025-09-07

**Authors:** William S Kim, Mari Iwasaki, Peiman Arghavanifard

**Affiliations:** 1 Internal Medicine, California Hospital Medical Center, Los Angeles, USA

**Keywords:** covid-19, delayed hemothorax, geriatric fall, geriatrics, ground level fall, post-traumatic hemothorax, rib fracture, video-assisted thoracoscopic surgery (vats)

## Abstract

Delayed hemothorax (DHTX) is a possible sequelae of thoracic trauma, especially in the setting of patients being treated with anticoagulation. We report the case of an 81-year-old Caucasian man with a DHTX presenting 14 days following an initial emergency department (ED) visit with multiple rib fractures due to a fall from the patient’s bed. Upon presentation to the ED a second time, the patient was hospitalized, tested positive for COVID-19, and on the second day of admission underwent video-assisted thoracoscopic surgery (VATS) without bleeding or other complications. This case illustrates that the initial weeks following thoracic trauma present a critical time window during which monitoring for complications such as spontaneous or DHTX can be beneficial, especially in patients being treated with anticoagulant medication. VATS is safe in patients who can tolerate single-lung anesthesia and provides a less invasive way to resolve the underlying pathology while providing comparable or even improved clinical outcomes, and should be considered when treating patients with hemothorax.

## Introduction

Rib fractures are particularly common in the elderly population, with comorbidities such as osteoporosis increasing the risk of even minor mechanical falls leading to injury and further complications [1]. Rib fractures can lead to hemothorax in the days and weeks following injury, a condition known as trauma-induced or delayed hemothorax (DHTX).

The detection and management of DHTX in patients with thoracic trauma are crucial due to its associated complications, including acute respiratory distress syndrome, sepsis, and empyema. The median time from initial trauma to DHTX assessment was 12 days, according to a retrospective study conducted using the National Readmissions Database [1]. Case reports of COVID-19 positive patients have also indicated that DHTX is a possible sequelae in patients being treated with anticoagulants [2-4].

Treatment options for DHTX include pleural drainage via chest tube placement, video-assisted thoracoscopic surgery (VATS), and open thoracotomy. VATS is reported to be employed in 10.9% of cases and shows advantages in reducing operative difficulty, contamination, and length of hospital stay, particularly when recommended within the first three to seven days of hospitalization [5]. More specifically, VATS results in 92% of patients being pain-free, 3% needing regular analgesics, 89% reporting full recovery, and 81% resuming normal life, against 50%, 33%, 45%, and 60% in open thoracotomy cases, respectively [6].

In this case report, we present a case of a COVID-19-positive elderly man presenting with DHTX 14 days following initial injury concurrently on a direct oral anticoagulant (DOAC). Anticoagulation was temporarily held, and DHTX was resolved with a VATS procedure conducted on the second day of admission.

## Case presentation

An 81-year-old man presented to the emergency department (ED) following a mechanical fall from the patient’s bed with multiple rib fractures. The patient had a past medical history significant for atrial fibrillation managed with Eliquis, congestive heart failure, bipolar schizophrenia, hypothyroidism, and hypertension.

**Figure 1:**
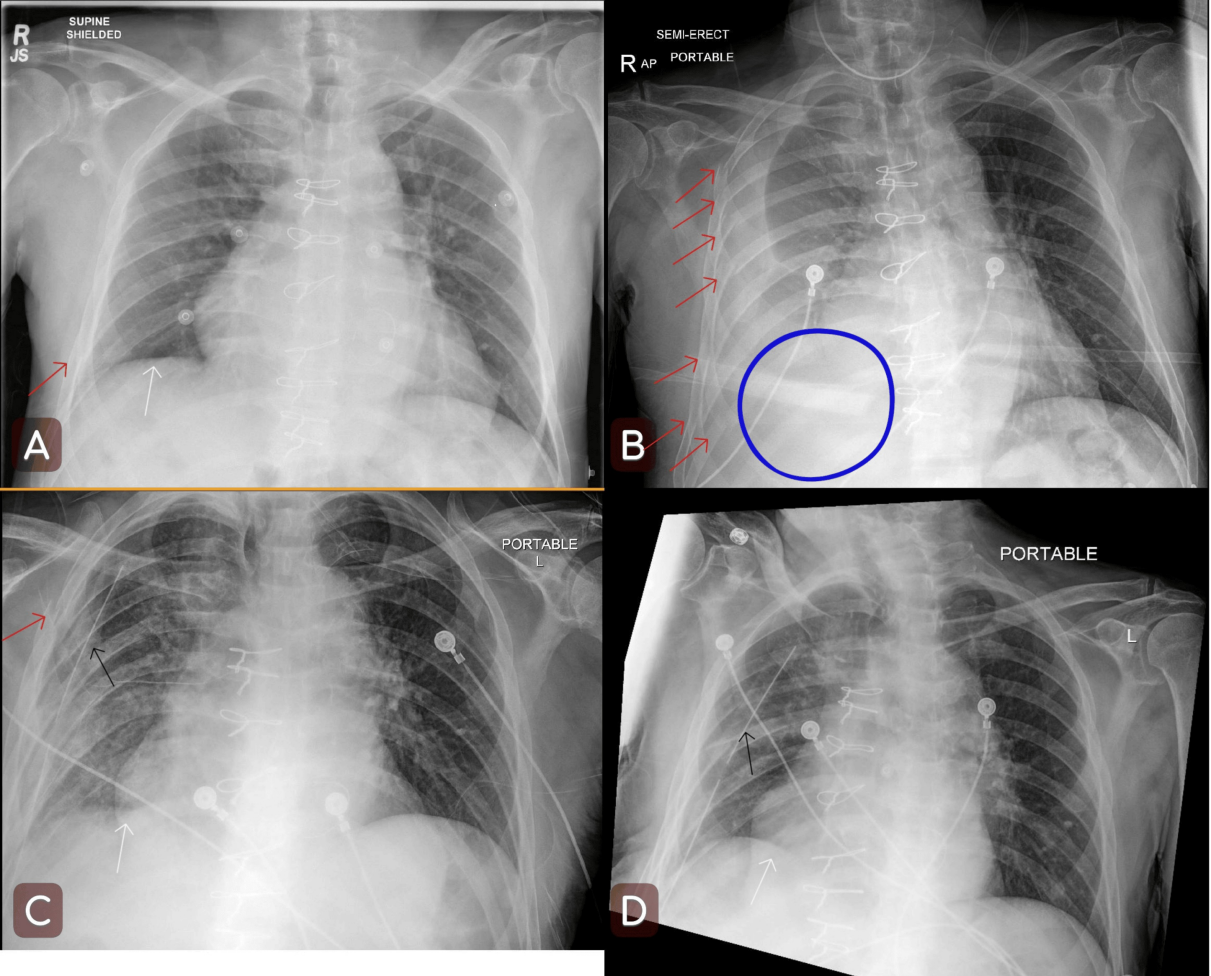
Serial Chest X-rays of the Patient Chest X-rays taken during the first ED visit, second ED visit, post-op day 1, and post-op day 8 (A, B, C, D). Rib fractures are marked with red arrows. Diaphragm outlines are marked with white arrows. Opacities in panel B representing pleural effusion are marked with a blue circle. The chest tube is marked using black arrows. Right lung borders in panel B are collapsed compared to other panels in the thorax, suggesting atelectasis.

Imaging studies taken in the ED showed cortical irregularity at the right lateral third rib (Figure 1, panel A), a chronically healed left posterior lateral fourth rib fracture, and mildly displaced fractures of the lateral eighth and seventh ribs, with subsequent imaging being unremarkable. The patient denied any head trauma or chest pain, was cleared by the trauma service, and discharged back to his skilled nursing facility the same day without any complaints.

Fourteen days later, the patient presented to the ED again, complaining of right-sided chest pain and shortness of breath. Chest radiographs and CT angiography with contrast were taken, and imaging studies showed trachea at midline, acute fractures of the right anterolateral third, fourth, fifth, sixth, and seventh ribs, acute fractures of the right posterolateral eighth, ninth, and 10th ribs, atelectasis of the right upper and middle lobes, complete consolidation of the right lower lobe, and a large right-sided pleural effusion with faint areas of contrast blush adjacent to right upper and lower fractures suggestive of hemothorax (Figure 1, panel B). Patchy left lower lobe atelectasis was also found, with no other significant findings in the left lung. Possible debris within the bronchi to the right lower lobe and possible central airway aspiration were also noted.

In the ED, the patient was alert but slightly hypothermic at 35°C, hypoxic at 86% on room air, and tachypneic at 24 breaths per minute. The patient also had a measured drop in hemoglobin levels from 12.6 g/dL on his first ED visit to 8.9 g/dL during presentation, an elevated white blood cell count of 11.4 thousand/uL, a B-type natriuretic peptide (BNP) level of 593.7, an international normalized ratio (INR) of 1.8, and a high lactic acid of 3.1 mmol/L. Additionally, the patient had a positive COVID-19 polymerase chain reaction (PCR) test and had a serum calcium level of 7.7 mg/dL. Subsequently, the patient was admitted for further evaluation and treatment. Full lab values at admission are provided in Table 1.

**Table 1:** Laboratory Values at Admission The table includes complete blood count (CBC), coagulation profile, general chemistry, cardiac, and microbiology. WBC: white blood cell count; RBC: red blood cell count; MCV: mean corpuscular volume; MCH: mean corpuscular hemoglobin; MCHC: mean corpuscular hemoglobin concentration; RDW: red cell distribution width; INR: international normalized ratio; PT: prothrombin time; PTT: partial thromboplastin time; BUN: blood urea nitrogen; CR: creatinine; A/G ratio: albumin/globulin ratio; ALT: alanine aminotransferase; AST: aspartate aminotransferase; hsTroponin-I: high-sensitivity troponin I; PCR: polymerase chain reaction; RSV: respiratory syncytial virus; SARS-CoV-2: severe acute respiratory syndrome coronavirus 2

Parameter	Value	Reference Range	Interpretation
WBC (×10^3^/µL)	11.4 ↑	4.0-11.0	Slightly elevated
RBC (×10^6^/µL)	3.09 ↓	4.5-5.9 (M), 4.1-5.1 (F)	Low
Hemoglobin (g/dL)	8.9 ↓	13.5-17.5 (M), 12.0-15.5 (F)	Low
Hematocrit (%)	26.5 ↓	38.8-50.0 (M), 34.9-44.5 (F)	Low
MCV (fL)	85.9	80-100	Normal
MCH (pg)	29	27-33	Normal
MCHC (g/dL)	33.7	32-36	Normal
RDW (%)	13.4	11.5-14.5	Normal
Platelets (×10^3^/µL)	309	150-450	Normal
INR (units)	1.8 ↑	0.9-1.2	High
PT (sec)	21.8 ↑	12.0-15.0	High
PTT (sec)	42 ↑	23-34	High
Sodium (mmol/L)	138	135-145	Normal
Potassium (mmol/L)	4.3	3.5-5.0	Normal
Chloride (mmol/L)	104	98-106	Normal
CO_2_ (mmol/L)	23	22-29	Normal
Anion Gap (mmol/L)	11	8-16	Normal
Glucose (mg/dL)	126 ↑	83-110	High
BUN (mg/dL)	22.00	8.40-25.70	Normal
Creatinine (mg/dL)	1.1	0.7-1.3	Normal
BUN/Cr Ratio	20.8	10-20	Slightly elevated
Calcium (mg/dL)	7.7 ↓	8.4-10.2	Low
Protein, Total (gm/dL)	5.1 ↓	6.40-8.30	Low
Albumin (gm/dL)	3.1 ↓	3.4-4.8	Low
Globulin	2	2.0-3.5	Normal
A/G Ratio	1.6	1.0-2.5	Normal
Bili Total (mg/dL)	0.7	0.2-1.2	Normal
ALT (Units/L)	24	0-55	Normal
AST (Units/L)	28	5-34	Normal
Alkphos (Units/L)	113	40.0-150.0	Normal
Lactic Acid (mmol/L)	3.1 ↑	0.5-1.9	High
B-natriuretic Peptide (pg/mL)	593.7 ↑	<=99.9	High
hsTroponin-I (ng/L)	23.0	0-35.0	Normal
Influenza A, PCR	Negative	Negative	Normal
Influenza B, PCR	Negative	Negative	Normal
RSV, PCR	Negative	Negative	Normal
COVID-19/SARS-CoV-2 PCR	Positive	Negative	Abnormal

During the hospital course, the patient was discontinued on Eliquis and was consulted with pulmonology, and was recommended for chest tube placement for drainage of the effusion the following morning, along with the recommendation of VATS with decortication if the effusion could not be fully drained. After discussion of the risks and benefits with the patient and family, consent was obtained for VATS, and the procedure was performed on the second day of admission.

The surgical report showed 750 mL of fluid drained from the chest. Follow-up chest radiograph showed near complete resolution of the right pleural effusion (Figure 1, panel C). Subsequent chest tube output following VATS is provided in Table 2. Serial chest radiographs were obtained during the remainder of the patient’s admission, and the patient was kept off Eliquis for the duration of the hospital stay. The patient was cleared of effusion on serial chest radiographs (Figure 1, panel D), had no further complaints or symptoms, and was discharged on the 13th day post-admission.

**Table 2:** Chest Tube Output Daily post-operative chest tube output following VATS. VATS: video-assisted thoracoscopic surgery

	Day 1	Day 2	Day 3	Day 4	Day 5	Day 6	Day 7	Day 8	Day 9
Output (mL)	540	810	250	150	40	50	210	120	30

## Discussion

DHTX is a possible complication following trauma to the thoracic region. In general, DHTX has also been reported to occur anywhere from 2 hours to 44 days after initial trauma [7]. DHTX has been seen in patients with a minor thoracic injury and commonly occurs when there is injury to the intercostal vessels or surrounding intrathoracic organs [8,9]. The number of rib fractures is a good marker for the likelihood of the development of DHTX in general. A cohort study reported that three or more rib fractures are the most sensitive risk factor known so far to predict pulmonary complications, including DHTX [10]. Additionally, some cases of DHTX have been associated with fractures or trauma to the spine [11].

In our case, the patient was positive for COVID-19 and was on a home medication regimen including a DOAC, and was recovering from a recent fall from his bed at his skilled nursing facility. Prior case reports have documented DHTX in COVID-19 positive patients treated with anticoagulants [2-4] and, separately, in patients admitted for chest trauma [1,8]. While prompt medical attention is paramount in elderly trauma cases, it is also important that ED resources are appropriately allocated and not overused [12]. According to ED trauma-response guidelines provided by the Washington Department of Health and the University of Arkansas Medical Center ED trauma manual, there seems to be a consensus that elderly patients with evidence of head trauma should undergo a CT head without contrast with urgent radiographic interpretation to rule out intracranial hemorrhage [13,14]. It is also important to determine whether and which type of anticoagulation a patient is on so that the medication can be held and reversal agents administered if appropriate [13,14]. This illustrates that the diagnostic work-up and decision to halt or continue anticoagulation in patients at-risk for DHTX is a nuanced problem that is currently best served at the discretion of the treating physician with evidence-based guidelines to direct treatment [12-15].

Lastly, according to a review article on hemothorax published in Clinical Pulmonary Medicine, definitive treatment for DHTX largely depends on how large the hemothorax is and whether the patient is hemodynamically stable [7]. For small DHTXs less than 300 mL, conservative treatment without intervention is a viable route. For larger effusions in hemodynamically stable patients, chest tube placement has been the most widely used intervention, but resolving the effusion via VATS has become increasingly widespread and effective [7]. Limitations of VATS, however, are that single-lung anesthesia is a requirement, and thus the patient must be hemodynamically stable to perform the procedure. Lastly, in cases where the patient is actively bleeding and is hemodynamically unstable, open thoracotomy is the most viable option [7]. If, however, the patient can be hemodynamically stabilized, VATS may be considered first [15]. Additionally, VATS has been found to be a viable treatment option in complicated COVID-19 cases [16].

## Conclusions

DHTX is a concern when treating patients with prior thoracic trauma, with the number of rib fractures having predictive value for the likelihood of DHTX, as mentioned in the discussion. This case illustrates that an elderly patient who initially presented in the ED for a fall-related trauma without any signs of pulmonary complications can develop hemothorax following discharge, putting the patient at risk for acute respiratory distress syndrome, sepsis, and empyema. Elderly patients on anticoagulation with a history of chest-related trauma are most likely at risk of developing pulmonary complications, as this case report illustrates. Further studies would be warranted to ascertain the best protocol for elderly patients on anticoagulation at-risk for DHTX, possibly in the setting of a multi-center ED-focused study on patients with thoracic trauma. Furthermore, an investigation as to whether a positive COVID-19 status has an additive effect on the risk factor for developing DHTX is merited.

## References

[1] Ouwerkerk JJ, Argandykov D, Gerban A (2023). Delayed hemothorax readmissions after rib fracture in blunt trauma patients. J Clin Orthop Trauma.

[2] Brogna B, Romano A, Tibullo L, Montuori M, Nunziata M, Russo G, Musto LA (2021). Rare findings of spontaneous hemothorax and small subpleural lung hematoma in a COVID-19 patient: a case report. Acta Radiol Open.

[3] Chai AL, El-Baba FM, Patel C (2023). Spontaneous hemothorax in a 48-year-old man with COVID-19 acute respiratory distress syndrome. Respir Med Case Rep.

[4] Mohan SA, Fadzaily ZS, Abdullah Hashim SH (2022). Spontaneous haemothorax in a patient with COVID-19. Case Rep Med.

[5] Mowery NT, Gunter OL, Collier BR (2011). Practice management guidelines for management of hemothorax and occult pneumothorax. J Trauma.

[6] Ben-Nun A, Orlovsky M, Best LA (2007). Video-assisted thoracoscopic surgery in the treatment of chest trauma: long-term benefit. Ann Thorac Surg.

[7] Zeiler J, Idell S, Norwood S, Cook A (2020). Hemothorax: a review of the literature. Clin Pulm Med.

[8] Chen CL, Cheng YL (2014). Delayed massive hemothorax complicating simple rib fracture associated with diaphragmatic injury. Am J Emerg Med.

[9] Sharma OP, Hagler S, Oswanski MF (2005). Prevalence of delayed hemothorax in blunt thoracic trauma. Am Surg.

[10] Chien CY, Chen YH, Han ST, Blaney GN, Huang TS, Chen KF (2017). The number of displaced rib fractures is more predictive for complications in chest trauma patients. Scand J Trauma Resusc Emerg Med.

[11] Okamoto K, Ichinose M, Hanaoka J (2018). Traumatic hemothorax due to chance fracture requiring emergency surgical management: a report of two cases. SAGE Open Med Case Rep.

[12] Pelaez CA, Spilman SK, Fuchsen EA, Semmens AD, Sidwell RA (2021). Trauma response for elderly anticoagulated patients: an initiative to reduce trauma resource utilization in the emergency department. J Trauma Nurs.

[13] (2015). Geriatric trauma clinical management guideline. https://medicine.uams.edu/surgery/wp-content/uploads/sites/5/2016/12/Geriatric-Trauma.pdf.

[14] (2019). Head injury in anticoagulated patients. https://doh.wa.gov/sites/default/files/legacy/Documents/Pubs/689160.pdf.

[15] Boersma WG, Stigt JA, Smit HJ (2010). Treatment of haemothorax. Respir Med.

[16] Bisagni P, Armao FT, Longhi M (2023). VATS in complicated COVID-19 patients: case series. Updates Surg.

